# Research on a Fault Feature Extraction Method for an Electric Multiple Unit Axle-Box Bearing Based on a Resonance-Based Sparse Signal Decomposition and Variational Mode Decomposition Method Based on the Sparrow Search Algorithm

**DOI:** 10.3390/s24144638

**Published:** 2024-07-17

**Authors:** Jiandong Qiu, Qiang Zhang, Minan Tang, Dingqiang Lin, Jiaxuan Liu, Shusheng Xu

**Affiliations:** 1 School of Mechanical Engineering, Lanzhou Jiaotong University, Lanzhou 730070, China; qiujd@mail.lzjtu.cn (J.Q.); 11220311@stu.lzjtu.edu.cn (Q.Z.); 11220295@stu.lzjtu.edu.cn (D.L.); 11230314@stu.lzjtu.edu.cn (J.L.); 11230295@stu.lzjtu.edu.cn (S.X.); 2 School of Automation and Electrical Engineering, Lanzhou Jiaotong University, Lanzhou 730070, China

**Keywords:** fault feature extraction, axle-box bearing of EMU, resonance-based sparse signal decomposition, variational mode decomposition

## Abstract

In light of the issue that the vibration signal from an axle-box bearing collected during the operation of an electric multiple unit (EMU) is seriously polluted by background noise, which leads to difficulty in identifying fault characteristic frequency, this paper proposes a resonance-based sparse signal decomposition (RSSD) and variational mode decomposition (VMD) method based on sparrow search algorithm (SSA) optimization to extract the fault characteristic frequency of the bearing. Firstly, the RSSD method is utilized to decompose the signal based on the obtained optimal combination of quality factors, resulting in the optimal low-resonance component with periodic fault information. Then, the VMD method is performed on this low-resonance component. The parameter combinations for both methods are optimized utilizing the SSA method. Subsequently, envelope demodulation is applied to the intrinsic mode function (IMF) with maximum kurtosis, and fault diagnosis is achieved by comparing it with the theoretical fault characteristic frequency. Finally, experimental validation and comparison are conducted by utilizing simulated signals and example signals. The results demonstrate that the proposed method extracts more obvious periodic fault impact components. It effectively filters out the interference of complex noise and reduces the blindness of setting weights on parameters due to human experience, indicating excellent adaptability and robustness.

## 1. Introduction

As an integral component of an EMU’s running gear [1], the role of the axle-box bearing pertains to the bearing and transmission. However, the adverse working environment exposes the axle-box bearing to a higher risk of damage and failure, which directly affects the operational reliability and safety of the EMU [2]. Therefore, exploring the fault diagnosis of axle-box bearings in EMUs holds significant implications. The analysis of vibration signals is one of the common methods to diagnose bearing faults [3]. When the bearing has an initial fault, the damage point will produce periodic impact force during the rotation process, which induces two types of vibration in the whole bearing system. One is the low-frequency vibration caused by the repeated impact of the damage point of the component on other components during operation. Its frequency is contingent upon the physical shape of the bearing and the rotation speed of the system. The other category involves the high-frequency vibrations in the bearing system induced by impulsive forces acting on the entire system [4]. When the severity of rolling bearing faults increases, its vibration signal exhibit noticeable nonlinearity and nonstationarity [5]. The signal is mainly composed of harmonic components, fault pulse components, and background noise. Different segments potentially exist with similar center frequencies and exhibit frequency-band overlap [6]. Therefore, the primary challenge of bearing fault diagnosis lies in effectively reducing the influence of background noise and harmonics and extracting fault characteristic frequencies from complex vibration signals.

To address the aforementioned challenges, extensive research has been conducted by scholars worldwide. This research has led to significant advancements in various techniques such as spectral kurtosis [7], short-time Fourier transform [8], resonance demodulation [9], wavelet transform [10], morphological filtering [11], sparse representation [12], minimum entropy deconvolution [13], and so on. The above signal processing methods initially use analytical expressions or adaptive methods to decompose a given signal into multiple different frequency bands. Feature extraction methods are then applied to each frequency band to extract fault feature quantities from them, implementing the diagnostic program. When the central frequencies of different components are superimposed together, multiple components in the vibration signal cannot be effectively separated through conventional methods such as frequency band decomposition or linear filtering. To address this issue, a novel signal processing approach called resonance-based sparse signal decomposition was proposed by Selesnick in 2011 [14]. This method integrates the morphological component analysis (MCA) and tunable Q-factor wavelet transform (TQWT) theory. Through different combinations of quality factors, the complex vibration signal can be decomposed into two components: the high-resonance component (composed of sustained oscillation components) and the low-resonance component (composed of transient impact components). In traditional applications of the RSSD method, the combination of quality factors typically needs to be set according to experience [15]. In order to enhance the adaptability of this method, scholars have employed optimization algorithms to explore the optimal combination of quality factors [16,17]. For example, Chen et al. [18] proposed a fault diagnosis method that combines RSSD with wavelet transform in response to the problem of effectively diagnosing the early fault of rolling bearings under strong background noise. This method defines the objective function as the kurtosis value of the low-resonance component and utilizes a genetic algorithm (GA) to explore the optimal combination of quality factors, aiming to enhance the decomposition performance and facilitate subsequent signal processing. Lu et al. [19] also employed the GA method to optimize the parameter combination selection for the RSSD method, effectively detecting weak faults in industrial equipment.

Although the RSSD method allows for the separation of the low-resonance component containing an impact component from the original signal, it still cannot completely eliminate the influence of noise, and the characteristic frequency of the impact component will be disturbed. To uncover and enhance fault features, additional methods such as empirical mode decomposition (EMD) [20], local mean decomposition (LMD) [21], and variational mode decomposition (VMD) [22,23] need to be employed. Gao et al. [24] processed the vibration signal by ensemble empirical mode decomposition and described bearing performance degradation by energy moment entropy. Based on the principle of quantum superposition, an enhanced long short-term memory model was designed for predicting early-stage bearing faults. To enhance the decomposition performance of the LMD method, Li et al. [25] focused on the improvement of the compound interpolation envelope technique to overcome the impact of nonstationary coefficient selection and achieved better results. Chen et al. [26] introduced a novel approach based on VMD and energy entropy to extract the fault feature vectors of bearings and compared this method with the method combining EMD and a db4 wavelet. The results demonstrate its superiority in terms of fault feature extraction. Xu et al. [27] integrated VMD with a convolutional neural network to directly process the raw vibration signal in an end-to-end manner, which can accomplish the fault diagnosis of rolling bearings without relying on manual experience and manual intervention.

However, both EMD and LMD exhibit issues with mode mixing and endpoint effects. Despite its advantages, including a simple algorithm, high computational efficiency, and the ability to effectively mitigate issues with mode mixing and endpoint effects, VMD requires presetting the number of decomposition modes and penalty factors before the decomposition process [28]. The setting of these parameters greatly affects the decomposition efficiency and effectiveness. In response to this problem, scholars have proposed some improved VMD methods for fault diagnosis. Dibaj et al. [29] determined the key parameters of VMD using an adaptive index and introduced a fine-tuning VMD for mechanical fault diagnosis. Nazari and Sakhaei [30] proposed a continuous variational mode decomposition method, which can extract modes continuously without knowing the number of modes and has higher robustness than VMD. Gu et al. [31] proposed an optimized VMD by selecting a combination of parameters using a genetic algorithm, with the objective of minimizing sample entropy. Chen et al. [32] initially applied the average autocorrelation method to denoise the vibration signals of an axle-box bearing. Then, aiming to minimize the reciprocal of the feature frequency energy ratio, they employed the Harris Hawk Optimization algorithm to select a combination of parameters for VMD. This approach enables the adaptive processing of vibration data and the stable extraction of axle-box bearing fault information. Aiming at the minimum modal overlapping component, Zhang et al. [33] sought the optimal solution for the main parameters in the multivariate variational mode decomposition algorithm through the Grey Wolf Optimizer. Additionally, they combined a multiscale convolutional neural network to identify feature vectors, enabling the diagnosis of complex composite bearing faults. This approach addresses the limitations of single-signal analysis methods in identifying features from multivariate data.

In summary, due to the complex operating environment of EMU axle-box bearings, the collected vibration signals typically contain a significant amount of noise and harmonic components. This poses great difficulty in extracting fault characteristic frequencies (especially in the early stages of faults), thereby impacting subsequent fault diagnosis tasks. Moreover, in practical applications, a specific method that is only applicable to a single operating condition is not economical. To enable the proposed method to cope with the fault feature extraction of various signals and mitigate the influence of human experience, the adaptive selection of optimal parameters is necessary. Enhancing the speed and accuracy of fault feature extraction methods is also beneficial for subsequent diagnostic processes. Based on this foundation, this study presents a method for the feature extraction of EMU axle-box bearing faults by combining resonance-based sparse signal decomposition optimized with the Sparrow Search Algorithm (SSA-RSSD) and optimized VMD. The main contributions of this study are as follows: (1) By combining the advantages of the RSSD and VMD methods and selecting different objective functions based on the characteristics of the processing results of these two methods, the impact of noise and harmonic components on fault feature extraction was significantly reduced; (2) utilizing the SSA method for the first time to optimize the selection of a quality factor combination exhibited a faster iteration speed and superior iteration results compared to the GA method; (3) the robustness of the proposed method was further explored by incorporating different noises. Through experiments conducted on simulated signal and bearing data from XJTU, the feasibility of the proposed method was validated. This method can avoid the suboptimal decomposition results caused by human error and effectively extract fault characteristic frequencies from complex bearing vibration signals. The superiority of the proposed method was demonstrated through a comparison with three advanced signal processing methods.

The structure of this paper is as follows: Section 2 presents the background and critical theories. Section 3 describes the proposed method. Section 4 establishes a validation of this method using simulated signals. Section 5 validates the feasibility of the proposed method in the XJTU bearing datasets. The final section concludes this study.

## 2. Background and Critical Theories

### 2.1. Problem Description of EMU Axle-Box Bearing Fault Diagnosis

**Figure 1:**
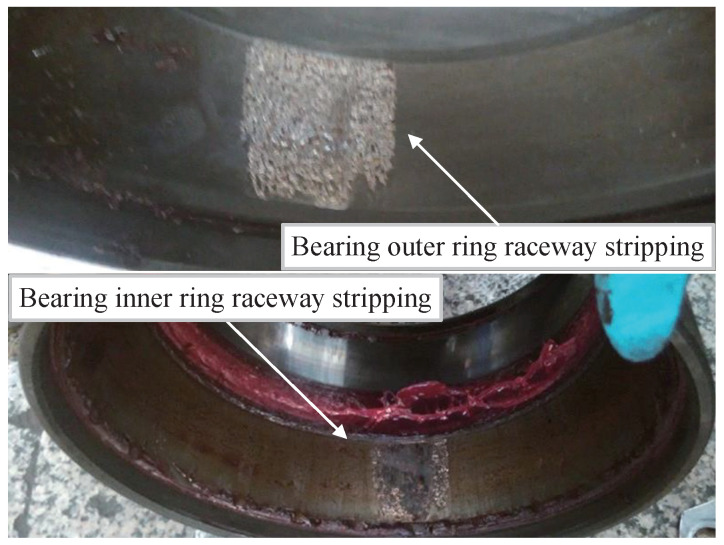
Bearing damage diagram.

Due to the influences of track irregularities, complex wheel–rail interaction, traction, and braking loads, the axle-box bearings of EMU may experience diverse types of failures under heavy loads, high speeds, and significant disturbances. Currently, EMU axle-box bearings commonly adopt rolling bearings that primarily comprise inner rings, outer rings, rolling elements, and cages [34]. In the actual maintenance process, the majority of failures occur in the inner and outer rings of the bearings, as shown in Figure 1. The failure modes of rolling bearings can be categorized into six distinct groups, namely, fatigue, wear, corrosion, electrical pitting, plastic deformation, and fracture. When a component of the bearing fails, the damaged point will collide with other surfaces, resulting in the generation of specific vibration signals and corresponding frequencies, known as fault characteristic frequencies. According to the description in reference [35], the amplitude of the failure frequency increases gradually as the bearing damage increases. In the absence of other signal interferences, after performing time–frequency transformation on the fault signal, a significant magnitude can be observed at the corresponding frequency in the frequency domain. However, during the actual operation of EMU, the entire system produces vibration signals that frequently overshadow the fault signals, causing indistinct fault characteristic frequencies [36]. Therefore, it is essential to conduct signal processing on the vibration signals to mitigate the influence of other signals and highlight the characteristic frequencies of fault signals.

### 2.2. Basic Flow of the Proposed Method

**Figure 2:**
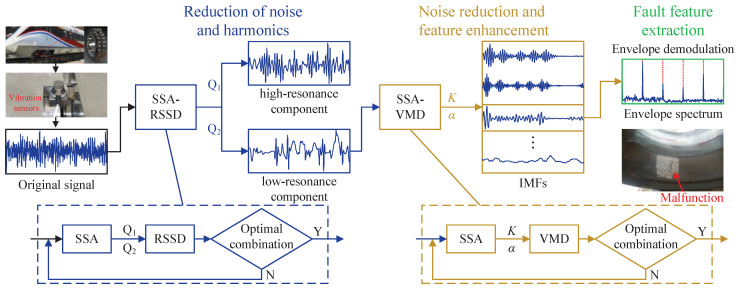
Flow chart of the proposed method.

To address the challenge of extracting the early fault features of axle-box bearings in EMU, the proposed method employs the process shown in Figure 2. The critical theories utilized are elaborated upon in the subsequent subsections in this section.

### 2.3. Resonance-Based Sparse Signal Decomposition

In the diagnosis of EMU axle-box bearing faults, different components of the vibration signal typically exhibit similar center frequencies, and the RSSD method offers an effective approach to the challenge of separating these different components. By combining the MCA and TQWT theory, the RSSD method is capable of effectively separating distinct components from the original signal. Firstly, in accordance with the oscillation property of the signal, the TQWT is employed to construct a wavelet basis function that aligns with the fault signal, enabling sparse representation of the signal. Subsequently, morphological component analysis is employed to establish an objective function, facilitating the efficient separation of diverse signal components [37]. The central objective of the RSSD method revolves around constructing tunable wavelets to decompose the original signal into distinct components comprising high resonance, low resonance, and residual resonance. Compared with the rational extended wavelet transform in the early resonance-based decomposition, the concept of TQWT is simpler; the radix-2 fast Fourier transform can be implemented more effectively. Simultaneously considering both the frequency and bandwidth factors allows this approach to effectively address the issue posed by overlapping components in a signal.

#### 2.3.1. Tunable Q-Factor Wavelet Transform

According to different quality factors, the TQWT can effectively disintegrate the complex signal into two distinct components: transient impact components and continuous oscillation components [18]. The quality factor, also known as the Q-factor, is represented by the ratio of the center frequency to the bandwidth, as shown in Equation (1).

(1) $$Q=\frac{f_{c}}{BW}$$

In this formula, $f_{c}$ denotes the center frequency (Hz), and BW represents the bandwidth (Hz) of a pulse signal.

**Figure 3:**
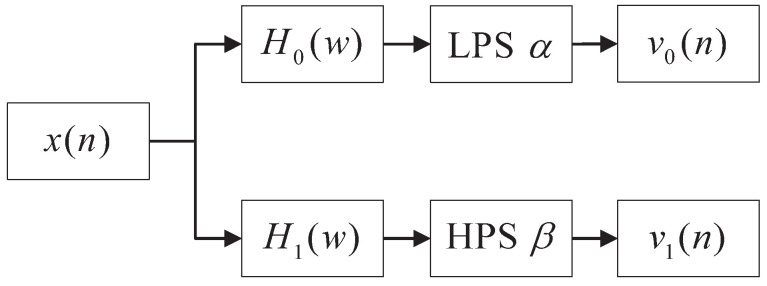
Dual-channel decomposition filter.

The Q-factor reflects the resonance characteristics of the signal, meaning that a higher Q-factor value indicates better signal frequency aggregation, and vice versa. TQWT can be achieved using a band-pass filter, as depicted in Figure 3 [38]. x(n) represents the original input signal. $H_{0}\left(\omega \right)$ and $H_{1}\left(\omega \right)$ represent the frequency response of the low-pass and high-pass analysis filters, respectively. LPS and HPS, respectively, denote low-pass scaling and high-pass scaling. $v_{0}\left(n\right)$ and $v_{1}\left(n\right)$ represent the sub-band signal obtained after decomposition.

The relationship between the low-pass scale factor α, high-pass scale factor β, Q-factor *Q*, and redundancy coefficient *r* is shown in Equations (2) and (3).

(2) $$\beta =\frac{2}{Q+1}$$

(3) $$\alpha =1-\frac{\beta }{r}$$

Based on the aforementioned equation, the values of α and β can be obtained through computation once the parameters *Q* and *r* of the filter bank are determined. We assume that the vibration signal *x* can be represented as the summation of the oscillating signal $x_{1}$, the transient impulse component $x_{2}$, the residual, and the noise *n*. To facilitate decomposition analysis, the RSSD method utilizes morphological component analysis, which can estimate or determine the components of $x_{1}$ and $x_{2}$ separately. The base functions $S_{1}$ and $S_{2}$ can be obtained via TQWT. If signals $x_{1}$ and $x_{2}$ can be sparsely represented by these base functions, their estimation can be accomplished through objective function minimization, as illustrated by Equation (4).

(4) $$\begin{array}{c} J\left(W_{1},W_{2}\right)={∥x-S_{1}W_{1}-S_{2}W_{2}∥}_{2}^{2}+\lambda_{1}{∥W_{1}∥}_{1}+\lambda_{2}{∥W_{2}∥}_{1} \end{array}$$

Among them, $W_{1}$ and $W_{2}$ are the transform coefficients of $x_{1}$ and $x_{2}$. $\lambda_{1}$ and $\lambda_{2}$ represent regularization parameters. To minimize the objective function *J*, the split augmented Lagrangian searching algorithm is employed to iteratively update the transform coefficients $W_{1}$ and $W_{2}$. Using this method, the estimation of high- and low-resonance components can be obtained as follows:

(5) $${\hat{x}}_{1}=S_{1}W_{1}$$

(6) $${\hat{x}}_{2}=S_{2}W_{2}$$

#### 2.3.2. Selection and Influence of Q-Factor Combination

In the RSSD method, the selection and combination of two Q-factors are based on the oscillatory characteristics of the analyzed signal, and the selection of parameter combinations significantly affects the decomposition results. Therefore, the adaptive selection of the optimal parameter combination for signal feature extraction holds significant importance. The high Q-factor is utilized to match the high-frequency vibration and background noise produced by the entire system during bearing operation, namely, the high-resonance component. The low Q-factor is employed to match the fault impact components of faulty bearings, namely, the low-resonance component. The selection of both parameters should not only reflect the approximate characteristics included in each component but also ensure the separation of overlapping parts with the same center frequency. The relationship between the Q factors $Q_{1}$ and $Q_{2}$ is described in detail in reference [14], which provides the equation for calculating their maximum inner product, as indicated in Equation (7).

(7) $$\rho_{\max}\left(Q_{1},Q_{2}\right)=\sqrt{\frac{Q_{2}+1/2}{Q_{1}+1/2}}$$

Suppose that $Q_{1}>Q_{2}$. If there is little difference between $Q_{1}$ and $Q_{2}$, both resulting components may exhibit similar characteristics to the original signal. In order to enhance the accuracy and reliability of signal decomposition, it is desirable to minimize the correlation between the two Q-factor values, that is, increasing the difference between $Q_{1}$ and $Q_{2}$. However, excessively high values of *Q* can lead to a mismatch with the oscillation characteristics in the high-resonance component, resulting in suboptimal decomposition results. Therefore, for the purpose of attaining a more optimal decomposition effect, it is essential to reasonably select a combination of Q-factors.

In practical applications, the value of the redundancy coefficient *r* is typically chosen between 3 and 4. When the value of *r* is not less than 3, the pass band of the level frequency response will not exhibit the “flat top” phenomenon discussed in reference [39] (where the frequency response within the pass-band sub-interval approximates a constant).

According to the signal data length *N*, the value of the decomposition level *L* can be determined, as shown in Equation (8).

(8) $$L\leq \left\lfloor \frac{\log(\beta N/8)}{\log(1/\alpha )}\right\rfloor$$

The selection of the decomposition level *L* has a profound impact on the ultimate result of signal decomposition. The center frequencies and bandwidths of each sub-band decrease as the decomposition level increases. A lower number of decomposition levels may lead to a loss of signal features, while a higher number of levels may result in overfitting or excessive refinement. Thus, it is essential to ascertain the suitable decomposition level by taking into consideration the specific application scenarios and decomposition results.

In summary, the selection of α and β exerts a substantial impact on the decomposition performance of RSSD. Therefore, it is crucial to adaptively optimize the two Q-values in order to enhance the decomposition performance of RSSD.

### 2.4. Variational Mode Decomposition

The VMD method can decompose signals into multiple modes based on their characteristics, thereby further attenuating the influence of noise and enhancing the fault characteristics. It is improved based on Wiener filtering and the Hilbert transform method. This method aims to decompose the input signal into *K* sub-signals, with a minimized sum of estimated bandwidths. The decomposed sub-signals can reproduce the input signal and have specific bandwidth sparse characteristics. The constrained variational problem can be formulated in the following manner [27]:

(9) $$\begin{array}{c} \min_{\left\{u_{k}\right\},\left\{\omega_{k}\right\}}\left\{\sum_{k}{∥\partial_{t}\left[\left(\delta \left(t\right)+\frac{j}{\pi t}\right)*u_{k}\left(t\right)\right]e^{-j\omega_{k}t}∥}_{2}^{2}\right\}\\ s.t.\;f\left(t\right)=\sum_{k}u_{k}\left(t\right) \end{array}$$

In this formula, $u_{k}\left(t\right)$ is the kth component of the original signal after variational mode decomposition; *t* is time; f(t) is the original signal; $\omega_{k}$ is the center frequency of each modal component; * is the convolution operation symbol; δ(t) is a Dirac function; *j* is an imaginary unit. To tackle Equation (9), Dragomiretskiy et al. [23] introduced the Lagrange multiplier λ(t) of the quadratic penalty function into the above equation, resulting in the transformation of the original equation into an unconstrained optimization problem. The correction equation with an augmented Lagrangian function is shown in Equation (10).

(10) $$\begin{array}{cc} L\left(\left\{u_{k}\right\},\left\{\omega_{k}\right\},\lambda \right) & ={∥f\left(t\right)-\sum_{k}u_{k}\left(t\right)∥}_{2}^{2}+\langle \lambda \left(t\right),f\left(t\right)-\sum_{k}u_{k}\left(t\right)\rangle \\ \; & +\alpha \sum_{k}{∥\partial_{t}\left[\left(\delta \left(t\right)+\frac{j}{\pi t}\right)*u_{k}\left(t\right)\right]e^{-j\omega_{k}t}∥}_{2}^{2} \end{array}$$

In this formula, α is the penalty factor.

The aforementioned variational problem can be solved by alternating the direction method of multipliers. After reaching a stopping condition, the iteration is ended, and *K* IMF components are obtained.

The problems in VMD are as follows: In the process of the VMD method, the number of decomposition modes *K* and penalty factor α need to be predetermined, which depends on artificial experience. When the value of *K* is excessively small, it may lead to under-decomposition, resulting in modal loss or modal aliasing. Conversely, if the value of *K* is excessively large, it may cause over-decomposition, manifesting as the emergence of false modes and significantly affecting the decomposition speed. The penalty factor α also has a certain influence on the decomposition effect. The unreasonable setting of α can notably prolong the computational time of the algorithm, leading to occurrences of modal aliasing and center frequency overlap, which will affect the decomposition effect [40]. Therefore, it is of significant importance to study the adaptive optimization of *K* and α under various working conditions to enhance the decomposition efficiency and decomposition effect of the variational mode.

### 2.5. Sparrow Search Algorithm

To improve the adaptability of the two aforementioned methods under various operating conditions, it is essential to utilize optimization algorithms to optimize the parameter combinations. The SSA method is capable of abstractly describing the foraging and anti-predation behaviors of the sparrow population, exhibiting advantages, such as strong optimization capabilities and a rapid convergence speed. For simplicity, Xue et al. [41] divided the sparrow population into producers, scroungers, and alarms and developed corresponding rules to establish mathematical models.

The position X of sparrow population is denoted by Equation (11):

(11) $$X=\left[\begin{array}{cccc} x_{1,1} & x_{1,2} & \cdots & x_{1,d}\\ x_{2,1} & x_{2,2} & \cdots & x_{2,d}\\ \vdots & \vdots & \vdots & \vdots \\ x_{n,1} & x_{n,2} & \cdots & x_{n,d} \end{array}\right]$$

In this formula, *n* represents the number of sparrows, while *d* refers to the dimension of the variables to optimize. The following equation represents the fitness of all sparrows, denoted as $F_{X}$.

(12) $$F_{X}=\left[\begin{array}{ccccc} f([x_{1,1} & x_{1,2} & \cdots & \cdots & x_{1,d}])\\ f([x_{2,1} & x_{2,2} & \cdots & \cdots & x_{2,d}])\\ \vdots & \vdots & \vdots & \vdots & \vdots \\ f([x_{n,1} & x_{n,2} & \cdots & \cdots & x_{n,d}]) \end{array}\right]$$

In this formula, *f* is the fitness of the individual. Producers play a pivotal role in searching for food and guiding the flow of the entire group, capable of exploring for food in a broader area. The update of the producer’s position during each iteration can be expressed by the following equation:

(13) $$X_{i,j}^{t+1}=\left\{\begin{array}{c} X_{i,j}^{t}\cdot \exp\left(\frac{-i}{\beta \cdot T_{\max}}\right),R_{2}<f_{\mathrm{ST}}\\ X_{i,j}^{t}+Q\cdot L,R_{2}\geq f_{\mathrm{ST}} \end{array}\right.$$

In this formula, $X_{i,j}^{t}$ represents the location information of the *i* th sparrow in the *j* th dimension; β represents a random number within the range (0, 1]; $T_{\max}$ represents the maximum number of iterations; *Q* denotes a random number following normal distribution; L is a matrix composed of elements with a constant value of 1; $R_{2}$ denotes the alarm value within the range (0, 1]; $f_{\mathrm{ST}}$ denotes the safety threshold within the range (0.5, 1.0].

When $R_{2}<f_{\mathrm{ST}}$, there is no predator in the vicinity, and producers transition in an extensive search mode. When $R_{2}\geq f_{\mathrm{ST}}$, the presence of predators is detected by some sparrows, so the producer will guide all sparrows to migrate to other secure regions to find food. Joiners follow producers in their search for food. The location update can be described as follows:

(14) $$X_{i,j}^{t+1}=\left\{\begin{array}{c} Q\cdot \exp\left(\frac{X_{\mathrm{worst}}-X_{i,j}^{t}}{i^{2}}\right),i>n/2\\ X_{p}^{t+1}+\left|X_{i,j}^{t}-X_{p}^{t+1}\right|\cdot A^{+}\cdot L,i\leq n/2 \end{array}\right.$$

In this formula, $X_{\mathrm{worst}}$ denotes the most unfavorable position in the current group; $X_{p}$ denotes the best position for producers to occupy; *A* represents a 1×d matrix, where the elements are randomly assigned as −1 or 1. In addition, $A^{+}=A^{T}{\left(AA^{T}\right)}^{-1}$. When i>n/2, the less adaptable scroungers are more likely to go hungry, and the sparrow needs to fly elsewhere to find food.

The initial position of alarms is generated randomly within the entire group, with its mathematical model presented in Equation (15).

(15) $$X_{i,j}^{t+1}=\left\{\begin{array}{c} X_{\mathrm{best}}^{t}+\beta \cdot \left|X_{i,j}^{t}-X_{\mathrm{best}}^{t}\right|,\;f_{i}\neq f_{g}\\ X_{i,j}^{t}+M\cdot \left(\frac{\left|X_{i,j}^{t}-X_{\mathrm{worst}}^{t}\right|}{\left(f_{i}-f_{w}\right)+\varepsilon }\right),\;f_{i}=f_{g} \end{array}\right.$$

In this formula, $X_{\mathrm{best}}$ represents the present globally optimal position; M denotes the direction of sparrow movement, which is a random number within the interval [−1, 1]; $f_{i}$ represents the fitness of the current sparrow; $f_{g}$ and $f_{w}$ represent the present global best and worst fitness values, respectively; ε represents an intermediate variable to avoid the zero-division error. To succinctly describe the behavior of sparrows, it was set that $f_{i}>f_{g}$ denotes that a sparrow has reached the edge of the group, and when $f_{i}=f_{g}$, this signifies that a sparrow has perceived the danger and should proceed towards the direction of others to reduce the risk.

## 3. The Proposed Method

### 3.1. The Objective Function of the Optimization Algorithm

It can be seen from Section 2.3.2 that the Q-factor reflects the signal resonance characteristics, and the value of the Q-factor increases with an increase in the signal resonance characteristics. In the traditional RSSD method, $Q_{1}$ and $Q_{2}$ are determined manually according to experience, which cannot guarantee the appropriateness of the Q-factor selection. To quantitatively characterize the features of low-resonance components in both the time and frequency domains, and to mitigate the influence of singular values, this study employs a comprehensive index. This is defined as the ratio between the normalized permutation entropy $H_{P}$ and frequency domain kurtosis value FK, denoted as HFK [42,43]. Equation (16) represents the calculation expression for HFK.

(16) $$HFK=\frac{H_{P}}{FK}$$

Permutation entropy is a metric that quantifies the complexity of chaotic time series with noise. It increases with the complexity of the signal, making it a powerful tool for assessing the complexity of one-dimensional time series data [44].

We set one time series, X={x(i),i=1,2,...,n}, the reconstruction matrix for which is obtained via phase space reconstruction, as shown in Equation (17).

(17) $$Y=\left[\begin{array}{cccc} x(1) & x(1+t) & ... & x(1+(m-1)t)\\ x(2) & x(2+t) & ... & x(2+(m-1)t)\\ \vdots & \vdots & ... & \vdots \\ x(j) & x(j+t) & ... & x(j+(m-1)t)\\ \vdots & \vdots & ... & \vdots \\ x(k) & x(k+t) & ... & x(k+(m-1)t) \end{array}\right]$$

In this formula, j=1,2,...,k, *m* denotes the embedding dimension, *t* represents the time delay, and *k* denotes the number of reconstructed components.

Each row in the matrix represents a reconstruction component, with these components arranged in ascending order, as shown in Equation (18).

(18) $$x\left(i+\left(j_{1}-1\right)t\right)\leq x\left(i+\left(j_{2}-1\right)t\right)\leq \cdots \leq x\left(i+\left(j_{m}-1\right)t\right)$$

In this formula, $j_{1},j_{2},...,j_{m}$ represents the index of the column, where each element of the reconstructed component is located.

A reconstructed component can obtain a symbol, as in Equation (19).

(19) $$S\left(l\right)=\left\{j_{1},j_{2},...,j_{m}\right\}$$

In this formula, it is stated that l=1,2,...,k and k≤m!.

In the *m* dimensional phase space, there exist multiple mappings, and the corresponding symbol sequences S(l) of different mappings have m! kinds. The probability of each sequence is $P_{1},P_{2},...,P_{k}$. The permutation entropy $H_{P}\left(m\right)$ of *k* distinct symbol sequences of time series x(i) is shown in Equation (20).

(20) $$H_{p}\left(m\right)=-\sum_{j=1}^{k}P_{j}\ln\left(P_{j}\right)$$

When $P_{j}=1/m!$, $H_{p}\left(m\right)$ has the maximum value, and the maximum value is ln(m!).

To facilitate analysis by mitigating the influence of singular values, $H_{p}\left(m\right)$ is normalized to Equation (21).

(21) $$H_{p}=H_{p}\left(m\right)/\ln\left(m!\right)$$

In this formula, $H_{P}$ represents the normalized permutation entropy.

For any time series, the normalized permutation entropy $H_{P}$ satisfies $0<H_{p}<1$. $H_{P}$ reflects the random degree of the time series, $H_{P}$ small represents time series rules, and $H_{P}$ large represents time series randomness.

The kurtosis value can effectively characterize the pulse characteristics of the signal. A higher value of kurtosis indicates a greater presence of pulse components in the signal, but it is susceptible to outliers. The frequency domain kurtosis criterion is employed to mitigate the influence of outliers on the kurtosis value in the data. For the signal y(t), the frequency domain kurtosis value FK is shown in Equation (22).

(22) $$FK=\frac{\frac{1}{n}\sum_{i=1}^{n}{\left(y_{i}\left(f\right)-\bar{y(f)}\right)}^{4}}{\left(\frac{1}{n}\sum_{i=1}^{n}(y_{i}(f\right)-\bar{y(f)}{)}^{2}{)}^{2}}$$

In this formula, *n* denotes the number of spectral points; y(f) represents the amplitude of the signal y(t) Fourier transform; $\bar{y(f)}$ denotes the average value of the amplitude obtained as a result of Fourier transform.

### 3.2. The Process of the Signal Decomposition Method Based on SSA-RSSD

The comprehensive index HFK integrates the strengths of normalized permutation entropy and frequency domain kurtosis, quantifying the characteristics of low-resonance components separated through the RSSD method in both the time and frequency domains. Consequently, the objective function is determined as the HFK of low-resonance components, and the minimum HFK is calculated using the SSA method, so as to obtain the best $Q_{1}$ and $Q_{2}$. The process of the signal decomposition method based on SSA-RSSD is as follows:

(1) According to experience, when utilizing the RSSD to process bearing fault vibration signals, the decomposition levels *L* of high- and low-resonance components are, respectively, set to 30 and 15, while the redundancy factor *r* is set to 3.5 for both components. The value range of the high Q-factor $Q_{1}$ is [3, 6], the value range of the low Q-factor $Q_{2}$ is [1, 3], and the initial value is set to $Q_{1}=3$, $Q_{2}=1$.
(2) The various parameters of the SSA method are then set, and, subsequently, the population is initialized by randomly selecting several groups within the feasible range of high and low Q-factors.
(3) The initial fitness is computed, and the position of the seeker is updated according to the early-warning value.
(4) The location of subscribers is then updated.
(5) According to the current minimum HFK, the fitness and optimal position are updated.
(6) Iterating steps (3) to (5), the position of the sparrow after reaching the maximum number of iterations is outputted, and the optimal values of $Q_{1}$ and $Q_{2}$ are determined under the current data conditions. If the convergence conditions are not satisfied, steps (2) to (4) are repeated until the convergence conditions are reached.
(7) Taking the combination of Q-factors optimized by the SSA as parameters, the RSSD algorithm is utilized to process the fault vibration signal, resulting in the extraction of high- and low-resonance components.

### 3.3. Variational Mode Decomposition Based on Sparrow Search Algorithm Optimization

The decomposition effect of the variational mode decomposition algorithm is significantly influenced by both the number of decomposition modes *K* and the penalty factor α. In the case of an excessively large value for *K*, the signal will be over-decomposed. Too small a signal will lead to insufficient decomposition. Therefore, the SSA method is utilized to optimize the parameter combination. As some IMFs do not contain fault characterization information in their envelope spectrum, despite having a smaller HFK, only the frequency domain kurtosis FK is utilized as the objective function. The maximum FK is calculated by the SSA method, thereby obtaining the optimal combination of *K* and α.

The steps of the signal decomposition and reconstruction method based on SSA-VMD are as follows:

(1) According to experience, the search range of *K* is established as [2, 10], the search range of α is set as [10, 1500], and the initial values are set to K=2, α=100.
(2) Set the parameters of the SSA method, and then initialize the population; that is, randomly select several groups in the feasible region of *K* and α.
(3) The initial fitness is computed, and the position of the seeker is updated according to the early-warning value.
(4) Update the location of subscribers.
(5) The fitness and optimal position are updated according to the current maximum frequency domain kurtosis.
(6) Iterate step (3) to (5) until reaching the maximum number of iterations, then output the final position of the sparrow, and determine the optimal values of *K* and α under the current data conditions.
(7) Taking the *K* and α optimized by the SSA as parameters, the VMD algorithm is employed to process the low-resonance component, and the IMF component with the maximum frequency domain kurtosis is obtained.

### 3.4. Fault Diagnosis Process

The SSA-RSSD method adaptively separates the optimal low-resonance component from the original vibration signal, consequently diminishing the influence of noise and harmonics. The SSA-VMD method adeptly identifies the IMF with the most fault information, thereby effectively mitigating noise interference and enhancing the fault feature information. The innovative approach integrates two methods and is suitable for early fault feature extraction and the diagnosis of EMU axle-box bearings in intricate vibration environments. A detailed description is provided below:

(1) Firstly, the original vibration signal is processed utilizing the SSA-RSSD method described in Section 3.2 to obtain the optimal low-resonance component with the minimal HFK.
(2) Secondly, the optimal low-resonance component acquired in step 1 is further processed utilizing the SSA-VMD method described in Section 3.3 to extract the IMF with the highest frequency domain kurtosis.
(3) Finally, the envelope spectrum of the optimal IMF is obtained through envelope demodulation, enabling the observation of fault characteristic frequencies within the envelope spectrum, thereby facilitating the completion of fault diagnosis.

## 4. Simulation Signal Verification

### 4.1. Construction of Simulation Signal

In order to preliminarily prove that the proposed method can be effectively applied to the fault feature extraction and fault diagnosis of the EMU axle-box bearing, the simulated signal is constructed according to the characteristics of the axle-box bearing. In light of the interference of significant noise during the operation of the axle-box bearing, two different types of noise are added to the simulated signal. The simulated signal y(t) consists of a harmonic component $x_{1}\left(t\right)$, impact component $x_{2}\left(t\right)$, random noise $s_{1}\left(t\right)$, and white noise $s_{2}\left(t\right)$. The expression is shown in Equations (23)–(25).

(23) $$y\left(t\right)=x_{1}\left(t\right)+x_{2}\left(t\right)+s_{1}\left(t\right)+s_{2}\left(t\right)$$

(24) $$x_{1}\left(t\right)=\left[1+\cos\left(2f_{r}\pi t\right)\right]\times \cos\left(2f_{z}\pi t\right)$$

(25) $$x_{2}\left(t\right)=e^{\left(-2\pi t\times gf_{n}\right)}\times \sin\left[\pi f_{n}\times \sqrt{1-g^{2}}\left(t_{0}-KT\right)\right]$$

In this formula, $f_{r}$ denotes the amplitude modulation frequency, $f_{z}$ denotes the carrier frequency, $f_{n}$ represents the natural frequency, *g* denotes the damping coefficient, $t_{0}$ represents the single-period sampling time, *K* represents the repetition number, and *T* represents the repetition period.

**Figure 4:**
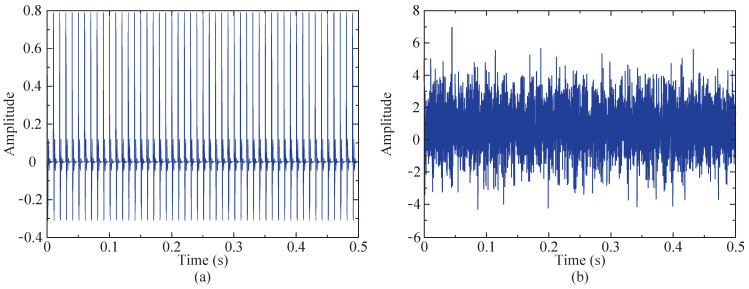
Time domain diagram. (**a**) Time domain diagram of impact component. (**b**) Time domain diagram of simulated signal.

The random value range of $s_{1}\left(t\right)$ is [0, 1], while $s_{2}\left(t\right)$ has a signal-to-noise ratio of −3 dB, the number of sampling points for y(t) is 4096, and the sampling frequency of the signal is $f_{s}$ = 8196 Hz. To facilitate a more intuitive observation of the changes in the signal before and after superposition, time domain diagrams were generated for both the impulse and simulated signals, as illustrated in Figure 4. The figure clearly indicates that the impulse signal has become submerged within other components.

**Figure 5:**
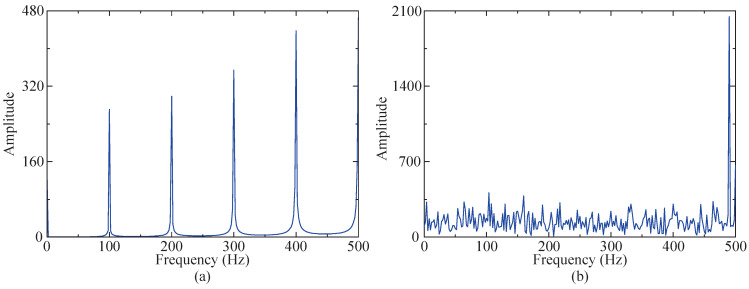
Envelope spectrum diagram. (**a**) Envelope spectrum of impact signal. (**b**) Envelope spectrum of simulated signal.

In order to more clearly compare and observe whether the characteristic frequency of the fault vibration signal is submerged by noise, envelope demodulation is applied to both the simulated signal and impact signal. The resulting envelope spectra are depicted in Figure 5 for further analysis. Figure 5a clearly reveals the characteristic frequency in the envelope spectrum of the impact signal. However, the envelope spectrum of the simulated signal does not show distinct impact signal characteristic frequencies. Its features include higher levels of bottom noise, larger peak amplitudes, and prominent amplitudes of harmonic signals (490 Hz). Therefore, the proposed method is employed to process the simulated signal, with the objective of accurately extracting the characteristic frequency of the impact signal.

### 4.2. Method Validation

**Figure 6:**
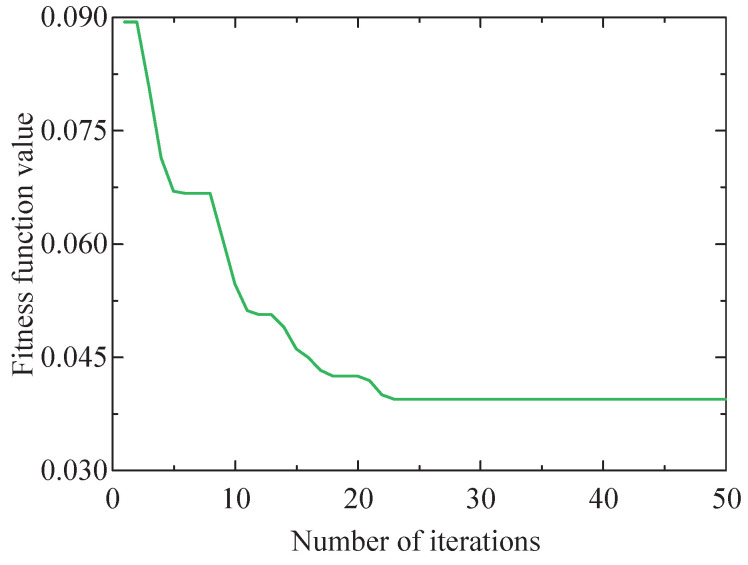
Iterative process of SSA method for HFK of the low-resonance component of simulated signal.

**Figure 7:**
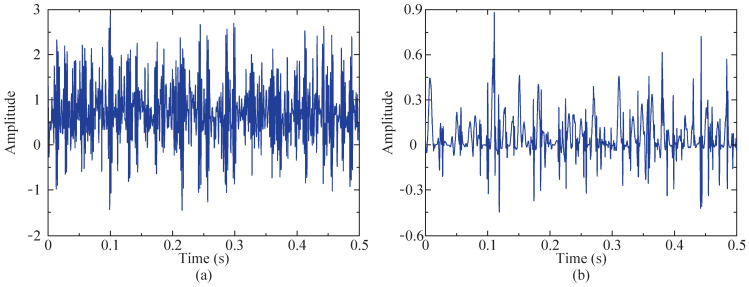
Results after SSA-RSSD method (time domain diagram). (**a**) High-resonance component of simulated signal. (**b**) Low-resonance component of simulated signal.

First, the SSA-RSSD method is utilized to denoise the vibration signal of the simulated faulty bearing, with the optimization objective function set as the minimum value of the HFK of the low-resonance component. The value of $Q_{1}$ and $Q_{2}$ is optimized by the SSA method to obtain the optimal combination. The range of the high Q-factor $Q_{1}$ is set as [3, 6], and the range of the low Q-factor $Q_{2}$ is set as [1, 3]. The iteration process of the SSA method for the objective function is shown in Figure 6. The figure illustrates that the optimal fitness value is observed as 0.03942489, with the corresponding values of the high and low Q-factors recorded as $Q_{1}=3.65$ and $Q_{2}=1.09$. Therefore, by setting this parameter combination, the simulated fault signal is subjected to decomposition using the RSSD algorithm, obtaining high- and low-resonance components, which are depicted in Figure 7.

Figure 7 demonstrates that the high-resonance component exhibits a waveform similar to the original simulated signal but more sparse, mainly including harmonic components and some noise and, notably, including random noise. The low-resonance component mainly includes the impact vibration component, but there are still some differences compared with the impact component of the original simulated signal.

**Figure 8:**
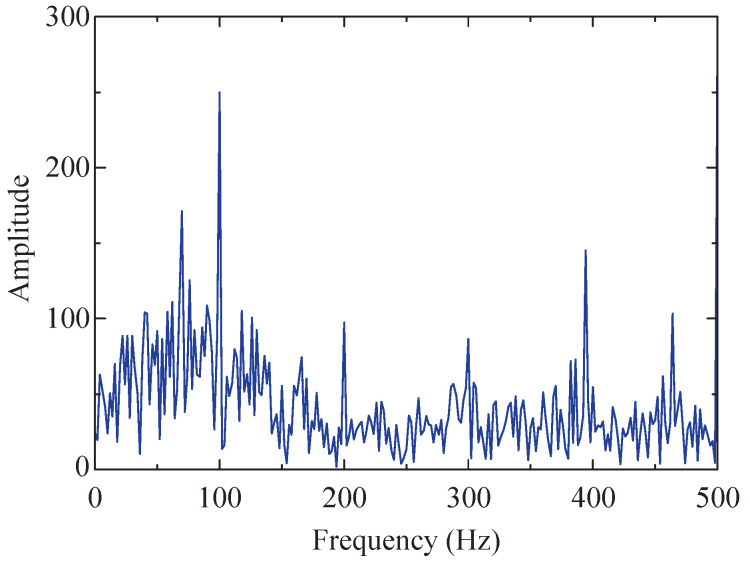
Envelope spectrum of the low-resonance component of simulated signal.

The envelope spectrum of the low-resonance component was obtained after applying envelope demodulation, as depicted in Figure 8. After performing SSA-RSSD processing and separating the low-resonance component from the signal, it is apparent that the high-amplitude part of the envelope spectrum is significantly reduced, but there are still interference peaks and bottom noises. This indicates that the SSA-RSSD method only achieves an initial noise reduction, and further processing is required to enhance the fault feature frequency.

**Figure 9:**
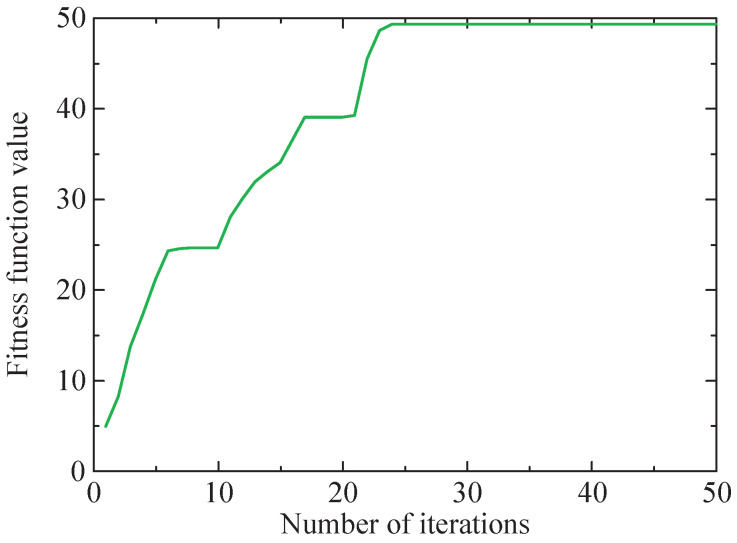
Iteration process of the SSA method with the maximum kurtosis value of IMF.

Then, the low-resonance component is processed using the SSA-VMD method, and the optimization objective function is the maximum kurtosis value of IMF. The iterative process curve of the SSA method is shown in Figure 9.

**Figure 10:**
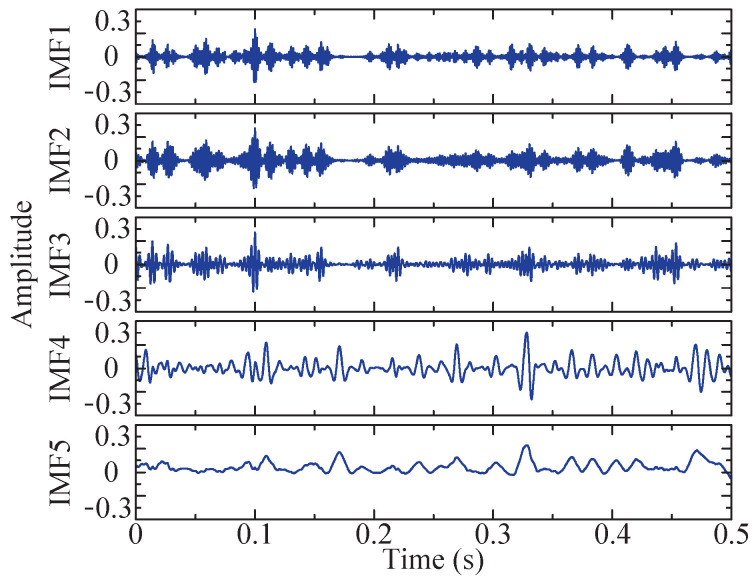
Time domain diagram of the five IMF components after VMD method.

**Figure 11:**
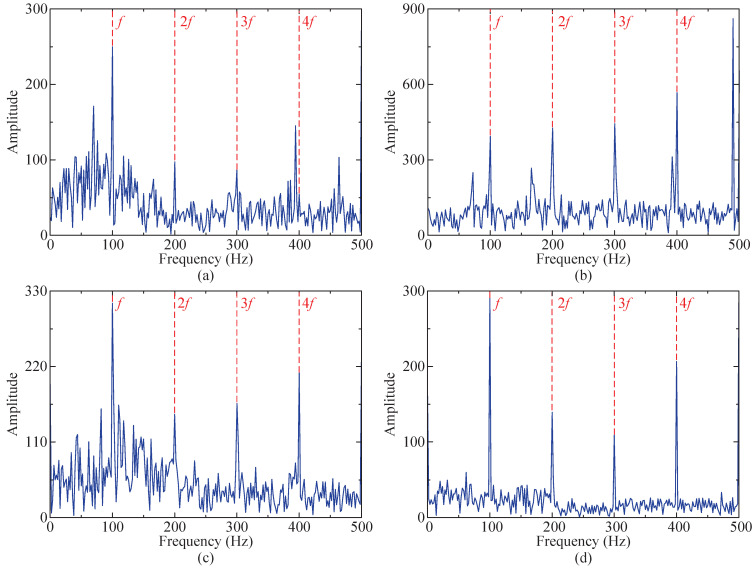
Results of four methods (envelope spectrum diagram). (**a**) SSA-RSSD method. (**b**) SSA-VMD method ([6, 1329], IMF4). (**c**) GA-RSSD method. (**d**) The proposed method.

The combination of the optimal *K* and α for VMD under the current conditions is determined by optimizing the SSA method. The current optimal fitness function value is 49.3473, and the corresponding optimal combination is [5, 1137], that is, K=5 and α=1137. Utilizing this parameter combination, five IMFs can be obtained through variational mode decomposition, as depicted in Figure 10. Among these IMFs, IMF3, exhibiting the largest kurtosis value, is selected for envelope demodulation analysis, and its corresponding envelope spectrum is obtained and denoted in Figure 11d.

From Figure 11d, the characteristic frequency *f* of the impulse signal and its multiples (2–4-times frequency) can be clearly observed, indicating that the proposed method effectively mitigates the influence of random noise and white noise, enabling the accurate extraction of fault characteristics from the impact signal submerged in the noise. Simultaneously, this study compares the optimization capabilities of the SSA-RSSD and GA-RSSD methods concerning the combination of Q-factors. The iteration results are presented in Table 1. The SSA method exhibits superiority over the GA method in terms of the iteration rate, iteration time, and iteration results, demonstrating its enhanced adaptability.

Furthermore, the proposed method is compared to the SSA-RSSD method, SSA-VMD method, and GA-RSSD method. The resulting envelope spectrum is illustrated in Figure 11. It is evident from the figure that by utilizing the SSA-VMD method solely for processing the simulated signal, some noise features will be enhanced, and the influence of harmonic signals cannot be mitigated. The outcomes obtained after signal processing employing the GA-RSSD method exhibit more interference peaks and stronger bottom noise compared to the SSA-RSSD method. Compared to the other three methods, the proposed method highlights fault characteristic frequencies more prominently and exhibits fewer interference peaks and lower bottom noise.

### 4.3. Robustness Testing

**Figure 12:**
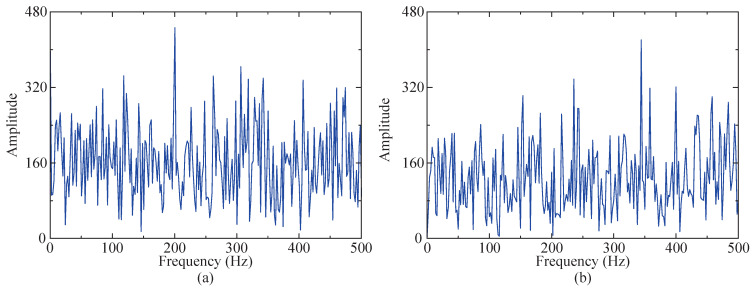
Envelope spectrum diagram of two noises. (**a**) Envelope spectrum of 5 times random noise. (**b**) Envelope spectrum of −1 dB white noise.

To further test the robustness of the proposed method in this study, the amplitude of random noise in the simulated signal was increased by five times, and the signal-to-noise ratio of white noise was adjusted to −1 dB, making the amplitudes of both noises closer to the impact signal after envelope demodulation. An envelope spectrum of the two noises is shown in Figure 12. The steps in Section 4.2 are repeated to process the new simulated signal.

**Figure 13:**
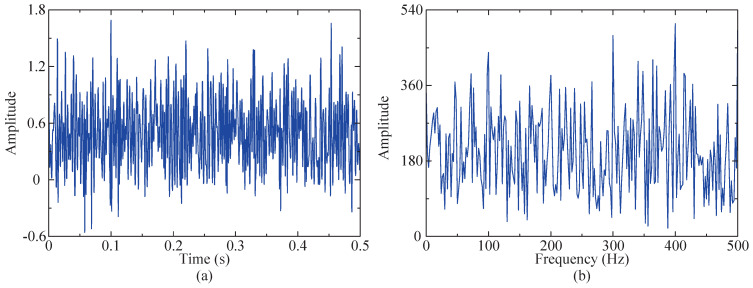
Results after SSA-RSSD method (new simulated signal). (**a**) Low-resonance component of new simulated signal. (**b**) Envelope spectrum of the low-resonance component of new simulated signal.

First, the SSA-RSSD method is utilized to obtain the optimal adaptation value of 0.064532053 after 32 iterations, with corresponding high and low Q-factors of $Q_{1}=4.83$ and $Q_{2}=1.25$. The low-resonance component and envelope spectrum of the new simulated signal are shown in Figure 13.

As depicted in Figure 13, even the optimal low-resonance component is still affected by new random noise, resulting in a slight upward shift in the overall signal. The time domain demonstrates a higher density of information compared to the low-resonance component before noise alteration. Although the envelope spectrum of the optimal low-resonance component can reveal the characteristics of the impact signal, it also exhibits a significant number of interference peaks and a heightened level of bottom noise. This indicates that the impact signal is more significantly affected by the introduction of new noise.

**Figure 14:**
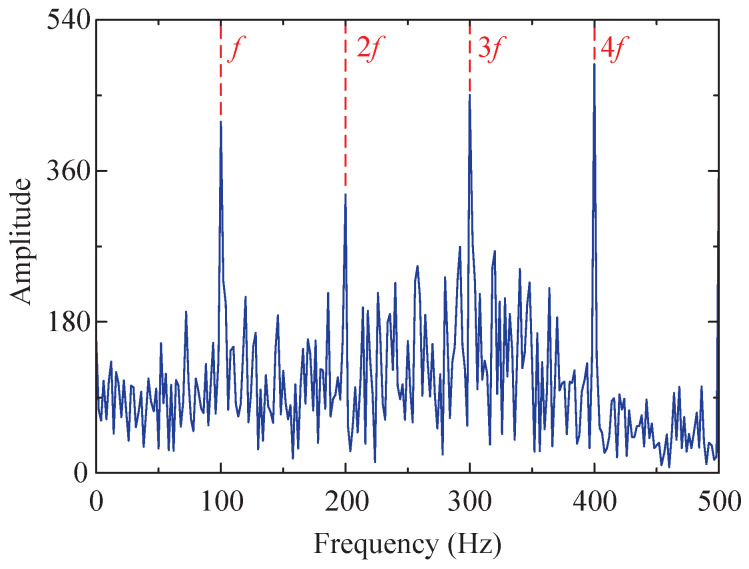
Envelope spectrum of IMF4.

Consequently, the SSA-VMD method was further employed to enhance the fault characteristics. Following 27 iterations, an optimal fitness function value of 23.8287 was attained, with the optimal combination of *K* and α being [7,937]. The envelope spectrum of IMF4, exhibiting the highest frequency domain kurtosis value, was obtained through envelope demodulation, as depicted in Figure 14. The figure reveals that despite the presence of some interference peaks, the characteristic frequency of the impact signal is effectively emphasized. The experimental results substantiate the robustness of the proposed method.

## 5. Example Verification

### 5.1. Data Declaration

**Figure 15:**
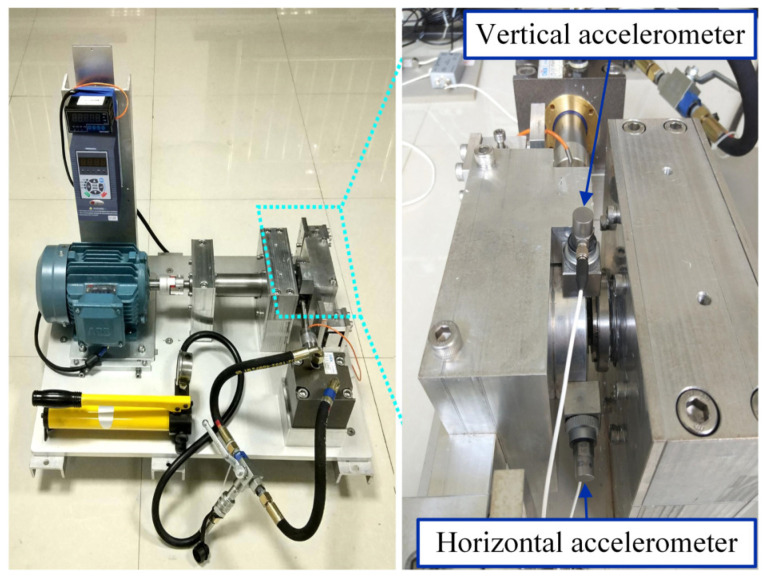
Bearing accelerated life test bench.

The XJTU-SY bearing datasets [45] were obtained from bearings during their operation until failure occurred. These datasets comprise vibration signals encompassing the complete lifespan of the bearings, from a healthy state to failure, and they serve to validate the effectiveness of the bearing fault feature extraction methods. Figure 15 shows the test bench used in these datasets. Two acceleration sensors are installed on the vertical axis and horizontal axis of the measured bearing seat. The test makes the bearing fail quickly by applying a load in the horizontal direction.

Vibration signals were recorded at a rate of 1.28 s every minute, with a sampling frequency of 25.6 kHz (32,768 sampling points). Table 2 shows information from the XJTU-SY bearing datasets. The datasets comprise tests conducted on 15 rolling bearings of the same model (LDK UER204) under three distinct operating conditions. These bearings have a rated dynamic load of 12.82 kN. The experiment was designed with three categories of operating conditions: Condition 1: a speed of 2100 rpm and a radial force of 12 kN; Condition 2: a speed of 2250 rpm and a radial force of 11 kN; Condition 3: a speed of 2400 rpm and a radial force of 10 kN. Each category of operating condition was tested with five bearings. The datasets also include comprehensive information pertaining to each tested bearing, encompassing the corresponding operating condition, total number of data samples, actual lifespan, and failure location. From the detailed information, it can be observed that outer-race failure accounts for two-thirds of the total failures, making it the most common location for bearing failures during operation.

**Figure 16:**
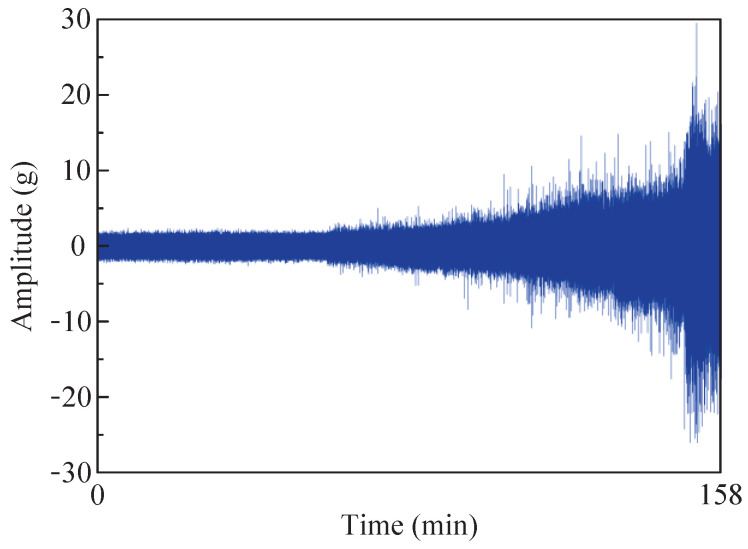
Time domain diagram of full-life horizontal vibration of fault bearing.

**Figure 17:**
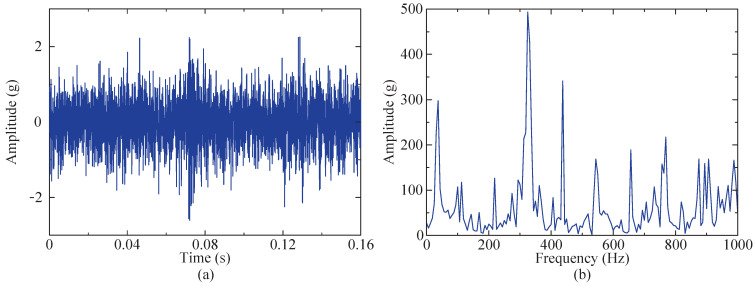
Initial analysis of instance signal. (**a**) Time domain of instance signal. (**b**) Envelope spectrum of instance signal.

Therefore, fault data from bearings 1–3 under the first operating condition were selected for experimental validation. Figure 16 shows a time domain diagram of the whole-life horizontal vibration of the bearing. The figure reveals that during the initial 60 min, the amplitude remained relatively stable, without significant variations. However, from 60 to 158 min, there was a noticeable and gradual increase in amplitude over time. Hence, it can be inferred that an early bearing failure occurred within this timeframe, so the data from the 50th minute were selected for analysis. To minimize the computational requirements, 4096 sampling points within this group of data were selected for fault feature extraction. Figure 17a,b present a time domain diagram and envelope spectrum of the selected data, respectively. According to the calculation formula for fault characteristic frequency, the outer ring of the bearing exhibits a fault characteristic frequency of 107.91 Hz, but this frequency is not prominently observed in the instance signal envelope spectrum. Therefore, the proposed method was employed to extract the fault characteristics.

### 5.2. Method Verification

**Figure 18:**
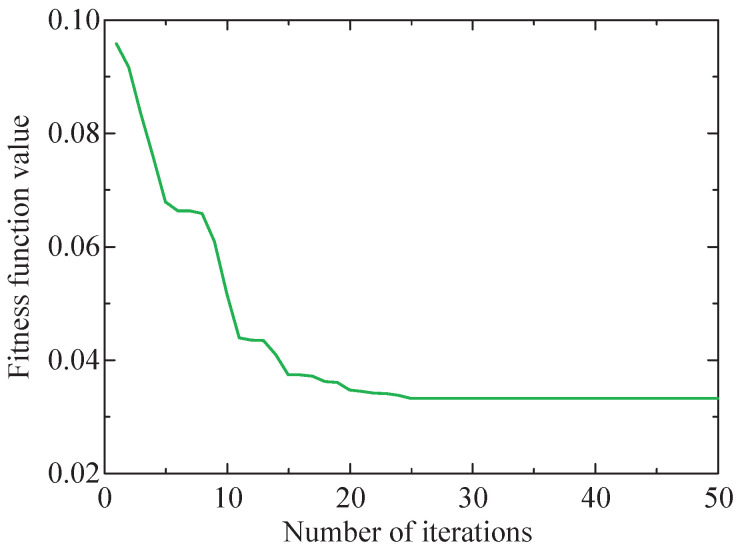
Iterative process of SSA method for HFK of the low-resonance component of instance signal.

Firstly, the SSA-RSSD method is utilized to denoise the vibration signal of the selected segment, with the optimization objective set as minimizing the HFK of the low-resonance component. The range selection of Q-factor combinations is consistent with the simulated signal. The iteration process of the SSA method for the objective function is shown in Figure 18.

**Figure 19:**
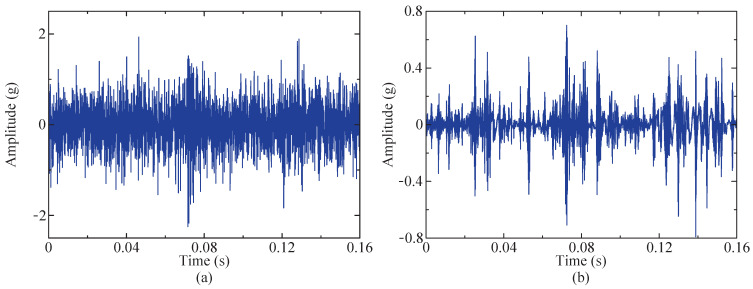
Results after SSA-RSSD method (time domain diagram). (**a**) High-resonance component of instance signal. (**b**) Low-resonance component of instance signal.

Figure 18 illustrates that the optimal fitness value is observed as 0.03323725, with the corresponding values of high and low Q-factors recorded as $Q_{1}=4.89$ and $Q_{2}=1.12$, respectively. Therefore, with this parameter combination set, the fault signal is subjected to decomposition utilizing the RSSD algorithm, obtaining high- and low-resonance components, which are depicted in Figure 19. The figure demonstrates that the high-resonance component mainly consists of high-frequency vibration components similar to the waveform of the instance signal, and the low-resonance component exhibits relatively lower amplitudes and sparser waveforms compared to the instance signal and contains transient impulse components, namely, fault impulsive components, in its waveform.

**Figure 20:**
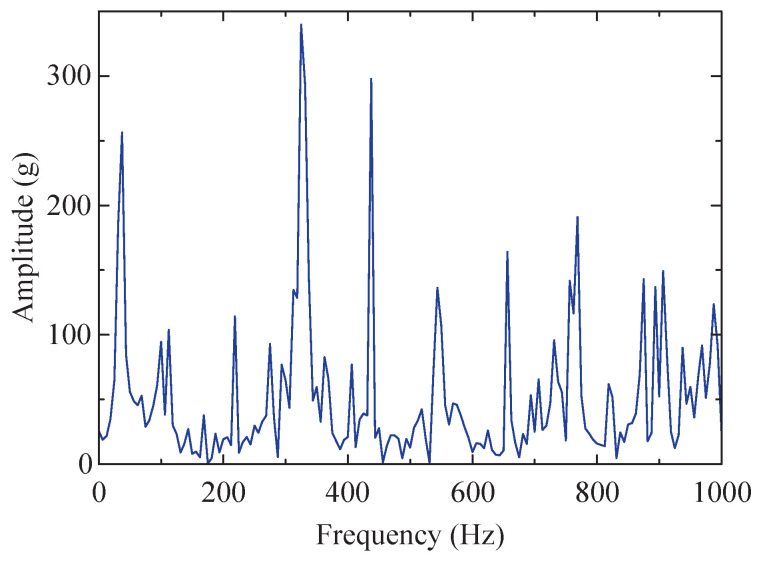
Envelope spectrum of the low-resonance component of instance signal.

Envelope demodulation was performed on the low-resonance component, and its envelope spectrum was obtained, as depicted in Figure 20. Compared to the envelope spectrum of the instance signal, after performing SSA-RSSD processing and separating the low-resonance component from the signal, it is apparent that the high-amplitude part of the envelope spectrum is significantly reduced, and the low-amplitude portion also experiences a decrease. However, interference peaks and some bottom noise still exist, which makes it difficult to clearly identify the fault characteristic frequencies. This indicates that the optimized RSSD algorithm achieved initial denoising, but further processing is required using the optimized VMD method.

**Figure 21:**
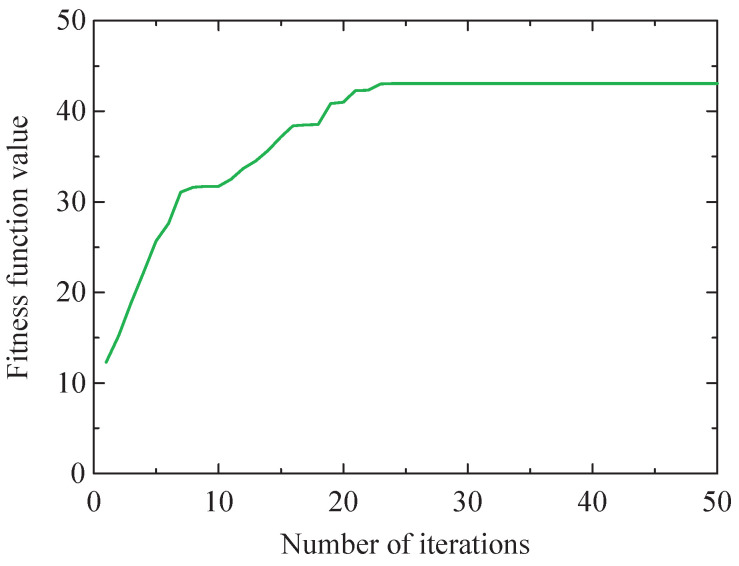
Iteration process of the SSA method with the maximum kurtosis value of IMF.

Then, the low-resonance component is processed utilizing the SSA-VMD method, and the optimization objective function is the maximum kurtosis value of IMF. The optimal *K* and α combination for VMD under the current conditions is determined by optimizing the SSA method. The iterative process curve of the SSA method is shown in Figure 21.

**Figure 22:**
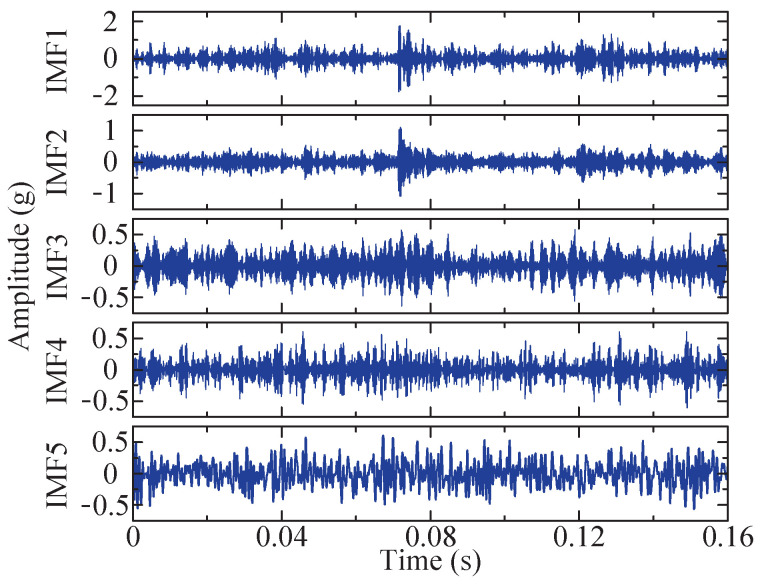
Time domain diagram of the five IMF components after VMD method.

The figure demonstrates that the optimal value of the objective function is determined to be 43.0822. Under this condition, the optimal *K* and α combination is [5, 1376]. Consequently, these two parameters are chosen, and five IMFs can be obtained through VMD. Their time domain diagrams are depicted in Figure 22.

**Figure 23:**
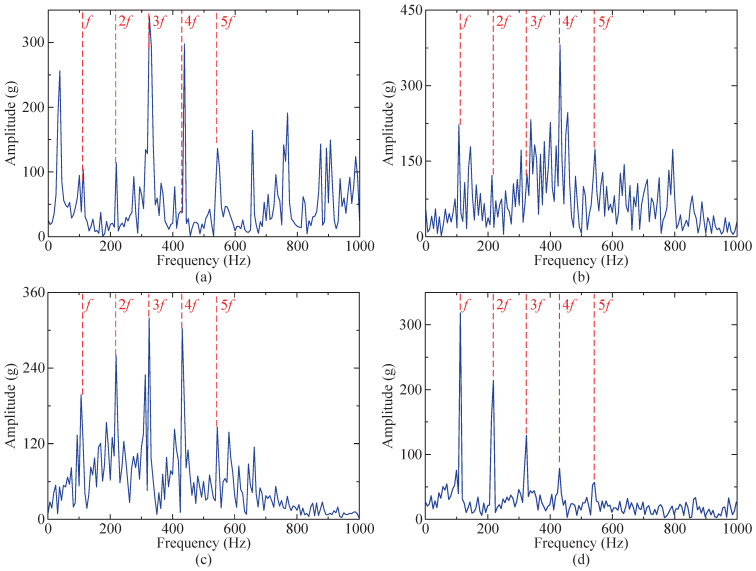
Results of four methods (envelope spectrum diagram). (**a**) SSA-RSSD method. (**b**) SSA-VMD method ([8, 536], IMF5). (**c**) GA-RSSD method. (**d**) The proposed method.

Among the obtained IMFs, IMF4 exhibits the largest kurtosis value and is therefore chosen for envelope demodulation analysis. Its envelope spectrum is depicted in Figure 23d, where the characteristic frequency *f* of the fault impact signal and its multiples (2–5-times frequency) are distinctly observable.

Similarly, the proposed method is also compared to the SSA-RSSD method, SSA-VMD method, and GA-RSSD method. The resulting envelope spectrum is illustrated in Figure 23. The diagram reveals that the fault characteristic frequency extracted by the proposed method exhibits greater prominence compared to the other three methods, exhibiting fewer interference peaks and lower bottom noise. The remaining comparative results are consistent with those obtained from the simulated signals.

In summary, the method proposed in this study effectively mitigates the influence of background noise and extracts the early bearing fault characteristic frequency submerged in the background noise. Furthermore, the optimization capabilities of the SSA-RSSD and GA-RSSD methods in terms of the combination of Q-factors were also compared for the instance signals. The iterative results are presented in Table 3. Consistent with the results for analog signals, the SSA method demonstrates stronger adaptability.

### 5.3. Robustness Testing

**Figure 24:**
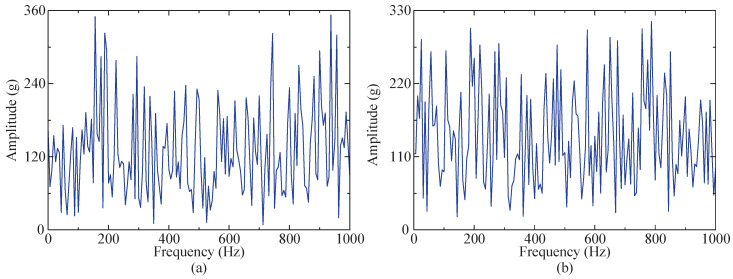
Envelope spectrum diagram of two noises. (**a**) Envelope spectrum of random noise. (**b**) Envelope spectrum of −2 dB white noise.

To further evaluate the robustness of the proposed method, random noise with a range of [0, 3.9 g] and −2 dB white noise were introduced to the instance signal. After envelope demodulation, both noises exhibited amplitudes closer to the fault characteristic frequency. The envelope spectrum of both noises is depicted in Figure 24.

**Figure 25:**
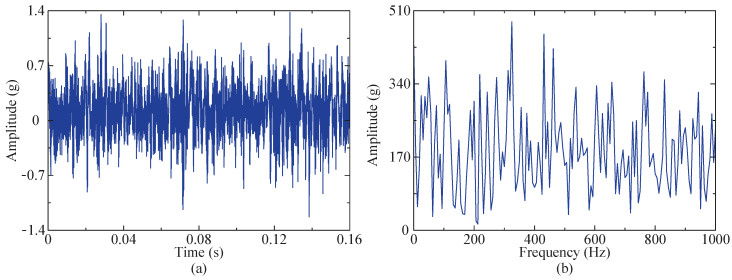
Results after SSA-RSSD method (new instance signal). (**a**) Low-resonance component of new instance signal. (**b**) Envelope spectrum of the low-resonance component of new instance signal.

The steps in Section 5.2 are repeated to extract fault features from the instance signal with added random and white noises. First, the SSA-RSSD method is utilized to obtain the optimal adaptation value of 0.056016074 after 34 iterations, with corresponding high and low Q-factors of $Q_{1}=5.75$ and $Q_{2}=1.36$. The low-resonance component and the envelope spectrum of the new instance signal are depicted in Figure 25. Similar to the robustness testing results on the simulated signal, the optimal low-resonance component of the new instance signal is also influenced by two types of noise.

**Figure 26:**
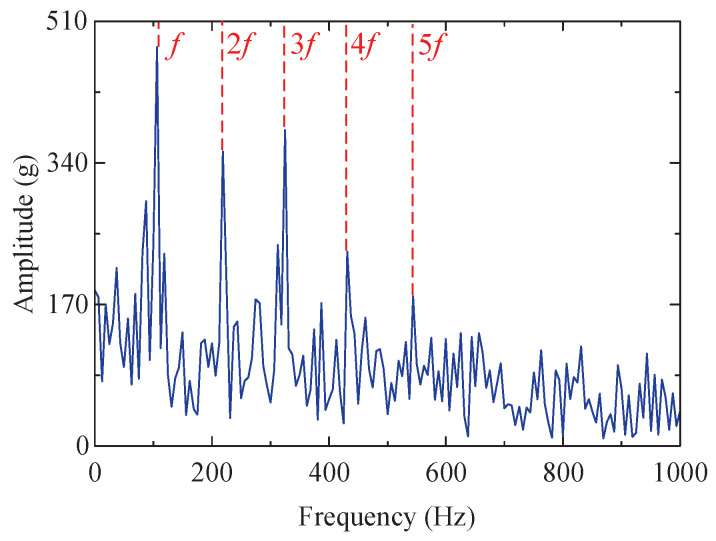
Envelope spectrum of IMF4.

The time domain plot shows a slight upward shift in the overall signal, with a slight increase in the amplitude. In the envelope spectrum, although the characteristic frequencies of the fault signals can be observed, multiple interference peaks and a higher level of bottom noise are present. The SSA-VMD method was further employed to enhance the fault characteristics, and the result is illustrated in Figure 26.

After 29 iterations, the SSA-VMD method obtained an optimal adaptation value of 30.3817, with the best combination of *K* and α being [8, 752]. The envelope spectrum of IMF4, exhibiting the highest frequency domain kurtosis value, was obtained through envelope demodulation, as depicted in Figure 26. The figure reveals that while there are still some interference peaks, the fault feature frequency is well pronounced. According to the experimental results of both simulated signals and instance signals, the proposed method demonstrates robustness.

## 6. Conclusions

Based on the signal analysis results of the aforementioned simulated signals and faulty bearing instance data, this study establishes the following conclusions.

(1) From the experimental results, it can be observed that the proposed method effectively extracts weak fault characteristic frequencies from complex signals and presents them clearly. The SSA-RSSD method is capable of isolating low-resonance components containing transient impact elements from the original signal, thereby reducing the influence of harmonics and noise. This method provides a preliminary extraction of the fault characteristics. Further enhancements and explorations of the fault characteristics can be achieved through the SSA-VMD method, leading to more accurate results.
(2) By employing the SSA method to optimize the parameter combinations, the influence of human subjectivity can be effectively reduced, thereby enhancing the adaptability of the proposed method. A comparative analysis of the results obtained via the proposed method with those of the other three methods was conducted. The results demonstrate that in terms of parameter optimization, the proposed method surpasses the GA-RSSD method, enabling faster and more accurate identification of the optimal parameter combination. In terms of highlighting weak fault characteristics and reducing the impact of noise and harmonics, the proposed method surpasses both the SSA-RSSD and SSA-VMD methods, achieving signal components with a higher signal-to-noise ratio.
(3) Through experiments, varying the noise in the simulated signal and introducing noise into the faulty bearing instance signal, the robustness of the proposed method is further demonstrated. From the results, it can be observed that despite the significant impact of various types of noise on the signal, the proposed method is still capable of extracting fault characteristic signals. Additionally, we found that as the impact of noise continues to increase, the proposed method gradually becomes unable to extract fault characteristic frequencies. In summary, the proposed method essentially achieved the research objectives, laying a foundation for subsequent fault diagnosis.

## Figures and Tables

**Table 1:** Comparison of iterative results of the two methods.

Method	Iteration Step	Computing Time	Optimal Fitness
GA-RSSD	25	109 s	0.04059189
SSA-RSSD	23	78 s	0.03942489

**Table 2:** LDK UER204 bearing parameters.

Parameter Name	Value	Parameter Name	Value
Inner race diameter/mm	29.30	Ball diameter/mm	7.92
Outer race diameter/mm	39.80	Number of balls	8
Bearing mean diameter/mm	34.55	Contact angle/(°)	0
Load rating (dynamic)/N	12820	Load rating (static)/N	66,500

**Table 3:** Comparison of iterative results of the two methods.

Method	Iteration Step	Computing Time	Optimal Fitness
GA-RSSD	26	113 s	0.03517653
SSA-RSSD	25	85 s	0.03323725

## Data Availability

The data that support the findings of this study are available at https://www.mediafire.com/folder/m3sij67rizpb4/XJTU-SY_Bearing_Datasets (accessed on 3 July 2023).

## References

[1] Zheng Z., Song D., Xu X., Lei L. (2020). A fault diagnosis method of bogie axle box bearing based on spectrum whitening demodulation. Sensors.

[2] Xu F., Ding N., Li N., Liu L., Hou N., Xu N., Guo W., Tian L., Xu H., Wu C. (2023). A review of bearing failure Modes, mechanisms and causes. Eng. Fail. Anal..

[3] Zhang M., Yin J., Chen W. (2022). Rolling Bearing Fault Diagnosis Based on Time-Frequency Feature Extraction and IBA-SVM. IEEE Access.

[4] David I.Z., Oscar T.P., Antonio V.G. (2019). Bearing fault diagnosis in rotating machinery based on cepstrum pre-whitening of vibration and acoustic emission. Int. J. Adv. Manuf. Technol..

[5] Wang L. (2023). Research on Bearing Diagnosis Method Based on Entropy Theory of Time Series Arrangement. Master’s Thesis.

[6] Yin C., Wang Y., Ma G., Wang Y., Sun Y., He Y. (2022). Weak fault feature extraction of rolling bearings based on improved ensemble noise-reconstructed EMD and adaptive threshold denoising. Mech. Syst. Signal. Process..

[7] Zhang X., Wan S., He Y., Wang X., Dou L. (2021). Teager energy spectral kurtosis of wavelet packet transform and its application in locating the sound source of fault bearing of belt conveyor. Measurement.

[8] Quinde I.R., Sumba J.C., Ochoa L.E., Vallejo Guevara A., Morales-Menendez R. (2019). Bearing fault diagnosis based on optimal time-frequency representation method. IFAC-PapersOnLine.

[9] Hao Y., Zhang C., Lu Y., Zhang L., Lei Z., Li Z. (2024). A novel autoencoder modeling method for intelligent assessment of bearing health based on Short-Time Fourier Transform and ensemble strategy. Precis. Eng..

[10] Han B., Song Z., Wei C., Zhou Y. (2023). Transient-extracting wavelet transform for impulsive-like signals and application to bearing fault detection. Meas. Sci. Technol..

[11] Zhou H., Yang J., Xiang H., Chen J. (2023). An adaptive morphological filtering and feature enhancement method for spindle motor bearing fault diagnosis. Appl. Acoust..

[12] Zhang W. (2018). Fault Diagnosis of Axle Box Bearing of High Speed Train Based on Sparse Representation. Master’s Thesis.

[13] Hou Y., Zhou C., Tian C., Wang D., He W., Huang W., Wu P., Wu D. (2022). Acoustic feature enhancement in rolling bearing fault diagnosis using sparsity-oriented multipoint optimal minimum entropy deconvolution adjusted method. Appl. Acoust..

[14] Selesnick I.W. (2011). Resonance-based signal decomposition: A new sparsity-enabled signal analysis method. Signal. Process..

[15] Chen X., Yu D., Luo J. (2012). Envelope demodulation method based on resonance-based sparse signal decomposition and its application in roller bearing fault diagnosis. J. Vib. Eng. Technol..

[16] Wang C., Li H., Ou J., Hu R., Hu S., Liu A. (2020). Identification of planetary gearbox weak compound fault based on parallel dual-parameter optimized resonance sparse decomposition and improved MOMEDA. Measurement.

[17] Zhang D., Entezami M., Stewart E., Roberts C., Yu D. (2018). Adaptive fault feature extraction from wayside acoustic signals from train bearings. J. Sound. Vib..

[18] Chen B., Shen B., Chen F., Tian H., Xiao W., Zhang F., Zhao C. (2019). Fault diagnosis method based on integration of RSSD and wavelet transform to rolling bearing. Measurement.

[19] Lu Y., Ding E.J., Du J., Chen G.C., Zheng Y. (2020). Safety detection approach in industrial equipment based on RSSD with adaptive parameter optimization algorithm. Saf. Sci..

[20] Wang W., Liu W., Lin C., Li M., Zheng Y., Liu D. (2023). Fault detection system of subway sliding plug door based on adaptive EMD method. Meas. Sci. Technol..

[21] Huang D., Ke L., Mi B., Zhao L., Sun G. (2018). A New Incipient Fault Diagnosis Method Combining Improved RLS and LMD Algorithm for Rolling Bearings With Strong Background Noise. IEEE Access.

[22] Zhang M., Jiang Z., Feng K. (2017). Research on variational mode decomposition in rolling bearings fault diagnosis of the multistage centrifugal pump. Mech. Syst. Signal. Process..

[23] Dragomiretskiy K., Zosso D. (2014). Variational Mode Decomposition. IEEE T. Signal. Process..

[24] Gao Z., Liu Y., Wang Q., Wang J., Luo Y. (2022). Ensemble empirical mode decomposition energy moment entropy and enhanced long short-term memory for early fault prediction of bearing. Measurement.

[25] Li X., Ma J., Wang X., Wu J., Li Z. (2020). An improved local mean decomposition method based on improved composite interpolation envelope and its application in bearing fault feature extraction. ISA Trans..

[26] Chen X., Yang Y., Cui Z., Shen J. (2019). Vibration fault diagnosis of wind turbines based on variational mode decomposition and energy entropy. Energy.

[27] Xu Z., Li C., Yang Y. (2020). Fault diagnosis of rolling bearing of wind turbines based on the Variational Mode Decomposition and Deep Convolutional Neural Networks. Appl. Soft. Comput..

[28] Wang J., Zhan C., Li S., Zhao Q., Liu J., Xie Z. (2022). Adaptive variational mode decomposition based on Archimedes optimization algorithm and its application to bearing fault diagnosis. Measurement.

[29] Dibaj A., Ettefagh M.M., Hassannejad R., Ehghaghi M.B. (2020). Fine-tuned variational mode decomposition for fault diagnosis of rotary machinery. Struct. Health Monit..

[30] Nazari M., Sakhaei S.M. (2020). Successive variational mode decomposition. Signal. Process..

[31] Gu J., Peng Y., Lu H., Chang X., Cao S., Chen G., Cao B. (2022). An optimized variational mode decomposition method and its application in vibration signal analysis of bearings. Struct. Health Monit..

[32] Chen C., Zhou L., Yang L. (2023). Axle box bearing fault diagnosis based on average autocorrelation and optimized VMD. J. Vib. Meas. Diagn..

[33] Zhang H., Shi P., Han D., Jia L. (2023). Research on rolling bearing fault diagnosis method based on AMVMD and convolutional neural networks. Measurement.

[34] Yu Y., Guo L., Chen Z., Gao H., Shi Z., Zhang G. (2023). Dynamics modelling and vibration characteristics of urban rail vehicle axle-box bearings with the cage crack. Mech. Syst. Signal. Process..

[35] Guo L., Yu Y., Chen Z., Liu Y., Gao H. (2024). Study on dynamic characteristics of urban rail transit vehicle considering faulty axle-box bearings under variable speeds. Mech. Syst. Signal. Process..

[36] Chen J., Hua C., Dong D., Ouyang H. (2023). Adaptive scale decomposition and weighted multikernel correntropy for wheelset axle box bearing diagnosis under impact interference. Mech. Mach. Theory.

[37] Yu G., Yan G., Ma B. (2022). Feature enhancement method of rolling bearing acoustic signal based on RLS-RSSD. Measurement.

[38] Selesnick I.W. (2011). Wavelet transform with tunable Q-factor. IEEE Trans. Signal. Process..

[39] Huang W., Sun H., Wang W. (2017). Resonance-Based Sparse Signal Decomposition and its Application in Mechanical Fault Diagnosis: A Review. Sensors.

[40] Zhou Q. (2021). Research on Diagnosis Algorithm and Generation Mechanism of Abnormal Noise for Automobile Engine. Ph.D. Thesis.

[41] Xue J., Shen B. (2020). A novel swarm intelligence optimization approach: Sparrow search algorithm. Syst. Sci. Control Eng..

[42] Fan J., Qi Y., Gao X., Li Y., Wang L. (2021). Compound fault diagnosis of rolling element bearings using multipoint sparsity–multipoint optimal minimum entropy deconvolution adjustment and adaptive resonance-based signal sparse decomposition. J. Vib. Control..

[43] Pan B. (2021). Research on Fault Diagnosis Method of EMU Axle Box Bearing Based on Optimized RSSD and VMD. Master’s Thesis.

[44] Bandt C., Pompe B. (2002). Permutation Entropy: A Natural Complexity Measure for Time Series. Phys. Rev. Lett..

[45] Wang B., Lei Y., Li N., Li N. (2018). A Hybrid Prognostics Approach for Estimating Remaining Useful Life of Rolling Element Bearings. IEEE Trans. Reliab..

